# Living with the Memories—Parents’ Experiences of Their Newborn Child Undergoing Heart Surgery Abroad: A Qualitative Study

**DOI:** 10.3390/ijerph17238840

**Published:** 2020-11-28

**Authors:** Ólöf Kristjánsdóttir, Annica Sjöström-Strand, Gudrún Kristjánsdóttir

**Affiliations:** 1 Faculty of Nursing, University of Iceland, Eirberg, Eiriksgata 34, 101 Reykjavík, Iceland; gkrist@hi.is; 2 Department of Health Sciences, Faculty of Medicine, Lund University, Box 157, 22100 Lund, Sweden; annica.sjostrom-strand@med.lu.se

**Keywords:** child, parent, congenital heart disease, heart surgery, qualitative research, content analysis, cross-border care, transitions

## Abstract

Parents of children with a congenital heart defect needing complex heart surgery are at high risk of developing health problems. One can assume that parents whose child undergoes heart surgery abroad will undoubtably face added and unique stressors and health vulnerabilities. The aim of this qualitative study was to explore the transition experiences of parents of children who underwent a complex heart surgery abroad as newborns 1–5 years ago. The qualitative content analysis methodology by Graneheim and Lundman was used. A purposive sample of twelve parents, whose child had undergone a heart surgery abroad, participated in face-to-face, semi-structured interviews. Interviews were transcribed and analyzed using inductive qualitative content analysis. The overarching theme of “living with the memories” emerged from parents’ experiences, emphasizing the long-lasting impact this stressful event had on their lives. These experiences were characterized by four main categories: (1) being in an unknown situation; (2) feeling connected; (3) wishing to be accepted; and (4) finding closure. The findings show that the transition of having a newborn child undergo heart surgery abroad superimposed on the expected parenthood. That parents need to feel connected and included as legitimate clients was highlighted in their stories of experienced vulnerabilities. The results highlight the need for interdisciplinary teams to support these vulnerable families, particularly with follow-up care.

## 1. Background

Having a child with a critical congenital heart defect (CCHD) is recognized as an extremely distressing experience for parents [1], inevitably leading to vast supportive care needs that begin at the child’s congenital heart defect (CHD) diagnosis and continue through surgery and childhood [2,3]. CHDs are the most common congenital abnormality among infants, with the global yearly prevalence estimated at about 1% [4]. Of these, 25% are predicted to have CCHD, which is a life-threatening condition requiring urgent heart surgery and intensive care at birth, followed by life-long medical follow-ups and often further surgery [5]. It is estimated that up to 30% of parents of children with CCHD develop post-traumatic stress disorder and 25–50% develop depression or anxiety [6]. This is problematic because a parent’s ability to care for their child diminishes if their own needs are not met [2,7]. Unfortunately, these parents continue to be at a higher risk of developing mental health problems due to a lack of social support, information, and mental support [2,3].

Transitions in hospital environments (e.g., from the pediatric intensive care unit (PICU) to a clinical ward) are known as challenging experiences for parents of critically ill children [8], including those with CCHD [9,10]. Under these circumstances, insecurity, fear, stress, and anxiety are reported to be experienced by parents due to, for example, the unfamiliarity and uncertainty of the new environments. Subsequently, these may result in various changes, impacting parental and child health and well-being [8,9,10]. 

Due to the highly specialized hospital treatments complex pediatric heart surgeries require, this medical service is not always available “at home”. In these instances, children may be referred overseas, where they receive state-funded cross-border care [11,12]. These unprecedented circumstances inevitably introduce heightened stress, and unique challenges to already vulnerable families. Currently, our understanding of state-funded cross-border care for children with CCHD and their families is limited. Given this dearth of knowledge, using a qualitative approach to the parental transition experience, is seen as a prerequisite for understanding the supportive care needs of these families. State-funded cross-border, or the transfer of patients between two health care systems where funding agreements are in place, is different from medical tourism, which implies patients traveling abroad to receive “out-of-pocket and third-party payments medical treatments” [13]. Both cross-border care and medical tourism involve the movement of patients and families between countries, cultures, and health care systems. This inevitably creates added stress, challenges, and burden. Even so, the contexts are very different, for example, the severity and acuity of newborn’s CCHD needing cross-border care is inevitably different from medical tourism. 

Scarce literature exists regarding child medical tourism [14,15]. Only three studies were found looking at parents’ experiences [16,17,18], all focusing on older children (not newborns) receiving cancer or blood-related treatments in the United States. Two include Spanish-speaking parents from low-income countries [16,17] and one English-speaking parents from a high-income country [18]. Overall, these studies showed that transitions into new cultural environments create stress and extra burdens on families [16,17,18].

According to Meleis’s transitions theory [19], transition is the movement from one life condition to another and involves an adaption to change. Transitions can be triggered by events that require geographical changes (e.g., relocation), where sudden and rapid adjustments to different environments are needed. This inadvertently increases individuals’ stress and depletes and disrupts their usual sources of support. Therefore, creating an imbalance which can affect individual’s vulnerability risk, health, and wellbeing [19]. The transitions experienced by parents of newborn children with CCHD needing heart surgery abroad, surely create an imbalance. Understanding these experiences is thus an important first step in developing quality health care for these families. Therefore, the aim of this study was to elucidate and describe parents’ transition experiences of having a newborn suffering from a heart defect and having to undergo heart surgery abroad. 

## 2. Methods

### 2.1. Design and Sample

A qualitative exploratory design, based on Graneheim and Lundman’s content analysis methodology, was chosen to describe parents’ experiences of their child undergoing heart surgery abroad [20,21]. This approach is well suited to capture a deeper understanding of a topic and to obtain a sense of individuals’ experiences from their own perspective. Here, the meaning of an experience is described in terms of what and how it is experienced, and surfaces from the actual text using manifest (categories) and latent (themes) content analysis. This type of analysis is preferable when populations or topics have limited research because it helps create new knowledge that can be woven into a new or existing theory [20,21]. To ensure quality, the consolidated criteria for reporting qualitative research (COREQ) guidelines were followed in preparing this manuscript, and are provided in Supplementary Table S1.

A purposive sampling method was used to select the participants based on whether parents identified as having a child (0–18 years old) with CHD who underwent heart surgery at a university hospital in the southern part of Sweden between 2014 to 2019. Participants were included if they were over 18 years, able to speak and understand Icelandic, and if no more than 5 years had passed since their child underwent heart surgery in Sweden.

### 2.2. Setting and Recruitment of Participants

Participants were initially recruited from a patient database at the largest hospital in Reykjavik, Iceland. After participating in a survey, parents were asked if the researchers could contact them again in the future for an interview study. Those who provided consent were contacted by the first author (Ó.K.) via email, during which they were informed about the current study’s goal and invited to participate. In total, 25 parents were invited to participate, and of these, 11 declined to participate. The final study sample consisted of 12 parents because two parents dropped out due to unforeseen events conflicting with the study.

Table 1 shows the sociodemographic and specific characteristics of the parents with regards to the travel and their child. Of the 12 participants, 7 were mothers and 5 fathers. Of these, three traveled abroad more than once, two had a child that underwent more than one surgery, one had a child that experienced severe complications and disability after surgery, and one parent had a child that died. All children were newborns when they traveled abroad for the first time for surgery. The time from child’s heart surgery abroad and parents interview ranged from 1-to-5 years. 

### 2.3. Data Collection

Data were collected between November 2019 and February 2020 via 12 individual face-to-face semi-structured interviews. The interview guide used was developed by the authors, informed by their previous work [22], confirmed in the authors group, and pilot tested both with one mother and during the first three interviews, which resulted in minor revisions. Each interview started with the broad question: “Can you please describe how you experienced having to travel to Sweden for your infant’s heart surgery?” Probing questions were asked to encourage participants to describe their experience as much as possible (e.g., “Could you please explain what you mean by that?”). Selected interview questions are listed in Table 2.

Before the interviews started, a written informed consent form was provided and signed by the participants, and the interviewer explained her role in the research. No prior personal relationship existed between any of the researchers and the participants. All authors are female, registered pediatric nurses, with clinical experience and a research background (Masters and Doctorate degrees).

All of the interviews were carried out by the first author (Ó.K.) and took place in accordance with the parents’ wishes. That is to say, 11 were conducted in a separate interview room at a university in Iceland, and one was conducted in a private room at the participant’s workplace. The interviews lasted between 45 and 136 min, during which time only the interviewer and interviewee were present in the room. At the end of the interview, parents were thanked for their time and willingness to share their stories. Parents did not receive any financial reward for participating in the study. 

### 2.4. Data Analysis

The data were analyzed using Graneheim and Lundman’s [20,21] content analysis approach. The codebook was developed from the initial analysis of the data (a posterior) using an inductive method. All interviews were audio recorded, transcribed verbatim by a professional transcriber, checked for accuracy, and translated from Icelandic to English to make them available to all of the authors. The data analysis process started with the three authors reading and rereading the transcribed text individually to familiarize themselves with the material. Next, the three authors created meaning units from the text that comprised of several words or sentences that helped answer the research question. The meaning units were then condensed and labeled with codes that were understood in relation to the context (i.e., an open coding process). Initial codes were created by the three authors, who also referred to the literature when creating the final coding list. These codes facilitated the identification of concepts. Next, the authors used a categorization process to identify categories and subcategories, followed by the identification of a theme. The authors used a process of reflection and discussion to agree on the codes, categories, and themes. This resulted in the creation of a coding tree; provided as a Supplementary Table S2. This process was well documented throughout.

### 2.5. Ethical Considerations

Each potential participant was provided with verbal and written information stating that: (a) participation was voluntary; (b) withdrawal from the study could be made at any time without reason; and (c) all data were confidential. Each participant was provided with a written informed consent form. All parents were informed that a registered psychologist would be available to them following the interview. Permission for the study was obtained from the National Bioethics Committee of Iceland (no. 18/204) and the Icelandic Data Protection Authority.

## 3. Results

The overarching theme of “living with the memories” emerged from parents’ closeness with their experiences, and its lasting presence in their lives. Although up to five years had passed since parents had traveled abroad for their child’s heart surgery, their memories and emotions were still vivid. In all instances, parents became emotional at some point during their interview. Table 3 illustrates the overarching theme, and the four categories and eight subcategories from which this theme emerged.

### 3.1. Being in an Unknown Situation

Parents memories of their child’s CHD diagnosis and heart surgery abroad revealed the superimposition of unanticipated and unwanted transitions on an expected transition of parenting a healthy and normal newborn. One father described this unexpected transition of the news vividly: “*just a complete shock to hear this just like that… [you thought] your child was going for a 15-minute routine test [at the hospital], but comes home 16 days later somehow… this is like [being on] a rollercoaster, you have no control. You just have to sit.*” (Father 5). In these unknown situations, parents’ lives were disrupted and somehow placed on hold. 

#### 3.1.1. Disrupted Life

All parents were shocked upon receiving the news of their child’s CHD diagnosis and stunned when told that their child needed to travel overseas for complex heart surgery. Parents previous envision and expectations of a normal parenthood were superimposed by previously unknown reality, or as one mother described, “*it was surreal when he [doctor] said: He must go to Sweden. We just stared at him: What are you talking about? … Go to Sweden? He was just born.*” (Mother 7). In this situation, parents’ lives changed instantaneously and often without any warning, leaving them feeling powerless. The chain of unanticipated changes and disruption was described as overwhelming and leaving parents at a loss for what to do, “*there was this overwhelming overflow of anxiety of all the tasks that lay ahead. You just asked yourself: What am I supposed to do now.*” (Father 2). 

In such strange and unknown situations, parents’ habitats were constantly changing, which forced them to adjust to new environments, with new rules and regulations. Hospital rules, such as only allowing one parent to stay overnight at the cardiac ward, having to share a room with other families, were felt to restrict parents’ ability to share habitats freely. Access to healthy food was also described as problematic, and sometimes it seemed that parents lived in a suitcase and sleep was a luxury: “*We got a room to stay in … Of course, she [the mother] just wanted to be next to him … I checked on them several times during the night. So, I did not sleep much, but it was still good to get a room and a shower, and we were there with the suitcases.*” (Father 5). Within these changing circumstances parents had to learn how to care for their child and themselves, in addition to maintaining a healthy relationship with their support systems in Iceland and Sweden. Creating an equilibrium within these situations was difficult.

Following the surgery and throughout childhood, the child’s illness continued to create disruptions in parents’ lives. This was exemplified by parents’ descriptions of their child’s continuous follow-up medical appointments, long-term therapies, and multiple surgeries abroad. The daily stress was evident in parents’ interviews, described by one mother as feeling like “*a balloon about to burst*” (Mother 3) following the aftermath, but today, three years post-surgery, her daughter goes “*weekly to physiotherapy and occupational therapy, then there are regular team meetings at the kindergarten… and then she naturally has regular follow-ups with her cardiologist and an orthopedic surgeon, an ophthalmologist, and the neurologist.*” (Mother 3).

#### 3.1.2. Life on Hold 

Amidst this turmoil of changes, parents simultaneously experienced the feeling that their lives were on hold. This feeling was mostly described as waiting for event to happen or milestones to be hit. The events and milestones identified as important, varied by parents. 

However, all parents talked about the day of the surgery as particularly hard. The anxiety and uncertainty before were followed by either joy and thankfulness when the outcome was positive, but grief, sadness, and anger when it was negative. The stress of waiting was perhaps most palpable in parents’ stories as ‘phone-call’: “*we just wandered around and kill time. And then he [surgeon] phoned when the surgery was over…Everybody jumped up when the phone rang and just Oh My God!*” (Mother 12). 

Following the surgery, parents then began waiting for the child’s recovery. In this regard, the PICU discharge was a significant milestone for parents. Although this time-point created anxiety, parents also understood how significant this milestone was for the child and the family as a whole. As described by one mother, “*it was a big step for him to get there [cardiac ward], it was just amazing, just for all the three of us to be together.*” (Mother 7).

Parents with other children at home expressed the stress of being separated from them. Some parents had never been away from their children before, and for the smaller children, explaining their absence was hard. Waiting to return home and reunite with their children, but not knowing at what date, was very distressing for the whole family: “*What was hardest was not knowing when we would go back home… every night she [the daughter at home] asked: Mama when are you coming home?*” (Mother 1).

This feeling of waiting continued for parents. Parents described anxiety as they waited for the child to hit developmental milestones and their worries as they waited for child’s next follow-up appointment with the cardiologist: “*But before the follow-up appointments, I become nervous. I always got a headache afterwards, sort of like de-stressing.*” (Mother 7). This waiting placed a strain on parents and put their lives on hold.

### 3.2. Feeling Connected

As parents moved through their unexpected and unwanted transitions of their child’s illness, supportive environment that provided comfort, familiarity, and connection, appeared in parents’ interviews as a situational condition that facilitated a healthy transition. In particular, the importance of feeling connected—to the child, the siblings, the spouse, the grandparents, the health professionals, and other families of peers—was evident. 

#### 3.2.1. Supportive Environment 

As parents prepared for the travel to Sweden, they asked questions and sought support from various sources. The pediatric cardiologist was described by all parents as their key person within the health care system that provided them support, being referred to as the “*rock*” (Father 4) and “*the main man*” (Father 5). Following parents return home, the cardiologist continued to be that key person and almost the only professional support. 

Spark, a nonprofit CHD parent organization, was mentioned by all parents as instrumental in preparing and supporting them throughout the journey. Their peer-to-peer program, parental Facebook groups, website, and staff was described as immaculate for seeking knowledge and information, as well as being a safe space in which parents were understood and able to envision the future. One father described Spark as a “*rescue squad*” (Father 10); a metaphor exemplified in mother’s rumination of her struggles leading up to the travel on of how to explain the situation, and say goodbye to her other daughters: “*we disagreed [she and her husband] … so I sought advice from other mothers [via Spark’s website] about how to best explain this to my daughters … I didn’t want to lie to them about where we were going.*” (Mother 3).

Parents always sheltered themselves by seeking closeness and support from the grandparents, who traveled abroad with the parents, except if they needed to care for the siblings at home. Both parents traveled with their child to Sweden in all instances, but siblings always stayed at home. But a supportive spouse was likely the most important source of support for all parents: “*We [he and his wife] were just going to solve this together and just hope for the best.*” (Father 2). 

As parents transitioned between countries, hospitals, and wards, it was important for them to build a safe and comforting habitat for the family. Here, the Ronald McDonald House was described as having the welcoming “home away from home” environment they needed. Furthermore, Icelandic countrymen in Sweden (i.e., grandparents, other families, and health professionals) were instrumental in creating familiarity and security: “*And our moms were also there with us. They often prepared food for us which was home-like and very helpful … as was having other three Icelandic families staying at the same time, also with children undergoing heart surgery.*” (Mother 11).

All parents felt that their child received quality health care both by health professionals in Sweden and Iceland, and were remembered for their competence and skills: “*The staff… was wonderful … I experienced so much professionalism and security while I was there and if I’d need to do this again, I’d insist on it being in Lund.*” (Mother 9). However, in seeking support for themselves, Icelandic health professionals working at the hospital in Sweden were frequently described as a significant source of support and help. Parents portrayed them as selfless and voluntary; these professionals provided the parents with what seemed like a crucial yet unofficial support network. They stepped in and touched many parents’ lives: “*And during that time… an Icelandic anesthesiologist, he… just stepped into a role, he had not needed to do this. I don’t know how we would have coped without having him there.*” (Father 6). The Icelandic health professional provided important security in their cultural familiarity, both in the care setting or as a support during medical meetings. It was clear that parents welcomed and preferred support from Icelandic health professionals because it helped them to better understand their situation: “*Because you knew nothing [prior to transferring to a cardiac ward] … we got some brochures and stuff… but there was an Icelandic nurse working on that ward, which was very nice. And she spoke to us in Icelandic about exactly everything.*” (Mother 12). 

#### 3.2.2. Handling the Experience 

Parents’ ability to cope following the news of their child’s CHD diagnosis and surgery abroad varied. Some parents seemed more resilient and quickly found their new normal. The unknown situation and challenges were seen as an assignment and personal qualities a facilitator: “*I was incredibly strong… I was so convinced that this would work out well.*” (Mother 1). Furthermore, parents took charge and became engaged in their healing. They created “*photo album*” (Father 2) of the journey, intentionally accepted help offered to them by the community, “*My boss said: Just go and as see a psychologist, just pick one you like…So we [him and his wife] went together to see a psychologist.*” (Father 10), as well as seeking out and asking for the help they felt they needed, “*I asked if it would not be a good idea if I talked to a psychologist or something…*” (Mother 1). 

Parents’ ability to face their fears, however varied. They struggled with the unexpected path their life had taken. Parents were worried about their child’s future and how their own future would be influenced by the illness, as illustrated by one father: “*If all goes wrong… then you’ll naturally go to a dark place … even if you decide not to hang yourself, you’re going to live with it, then life is still useless, so you’re just such a passenger.*” (Father 10). 

Knowledge and skills helped parents face the fear and handle their experiences. The act of knowing symbolized a need for control and hope. Parents’ need for knowledge varied, however. Some sought a deeper understanding of their situation, while others felt that too much information created fear and anxiety. Parents described how their own education enabled them to seek and gain resources. However, a lack of knowledge (e.g., of the Swedish language) created vulnerability and a feeling of disconnect. One father explained this when he said: “*Some of the Swedish nurses, just didn’t want to speak English … they just avoided talking [to us].*” (Father 4). Further, knowledge and information were often described in opposition. For example, information was provided or not received, or was helpful or paradoxical. When information contradicted parents’ hopes for a normal future for their child, it was described as threatening, even when signs of their child’s permanent disability were evident.

Talking about their experiences was a coping mechanism used by all parents. However, their readiness to share this experience varied, and some needed a long time before they were ready, “*Yes, at that time there were no tears… I really do not remember much from this time. And it was not until after a year had passed [since the heart surgery abroad] that I could talk about it.*” (Mother 11). Memories also emerged, that parents did not want to talk about. One father described how he wanted to forget some memories: “*[There are] unspeakable things I’ve… forgotten and blocked out, this is somehow such a tribulation*.” (Father 6).

After returning home, some parents sought help from professionals to handle their experiences and help them to recover. No parent, however shared memories of faith or religion in their coping. Furthermore, with the exception of walking, physical or spiritual activities were seldom mentioned by parents as a way of coping, although one father found it helpful: “*I started to meditate… sometimes I did yoga… I started to run long distance.*” (Father 10).

### 3.3. Wishing Being Accepted

Parents situational conditions could also inhibit a healthy transitional experience. In the interviews, not being seen as a legitimate client, emerged as an important inhibitor. Overall parents did not feel accepted, neither by the Icelandic or Swedish health care systems. They expressed a need to be accepted as a legitimate client, meaning both as a parent—with continuous and multiple parenting roles, and as a client—with complex health care needs. When overlooked, parents’ vulnerabilities emerged, and their risk of developing traumatic experiences seemed to increase. 

#### 3.3.1. Parenting Roles and Needs

Parents felt it was important for them to fulfill their parenting roles of presence and caretaking as best they could throughout the child’s traveling and hospitalization. If overlooked by health professionals, parents described feeling distressed and dismissed: “*I found it very difficult that someone [at the PICU] could just drive me away from my child… that all of a sudden [somebody could] just [say]: You can’t be with your daughter, now you just have to leave.*” (Mother 3). 

At the same time, the extremely stressful situations, and environments that parents found themselves in made parenting a challenge. Understanding how best to care for their child was not only difficult but also frightening: “*We just sat with him [in the PICU], which was extremely strange and surrealist to be sitting there and he was attached to 300 lines and all kinds of drugs and everything is beeping, and you do not know what is actually happening. That was a strange life experience. And you are like, should I be here or not? You just do not know how to behave.*” (Mother 1).

Parenting also involved creating relationships with health professionals. Here transparency and honesty about the child’s condition was a fundamental component of trust building. When parents were not included in the decision making concerning their child, as described by one father regarding his son’s critical condition, they felt as if their parental rights were being violated: “*You’d never keep such information [CHD condition severity] from a patient, you’d tell him exactly what the situation was. And I think it should be exactly the same for parents.*” (Father 6).

Furthermore, the disruption of the illness put a strain on spousal relationships. Parents described disagreements in their understanding of the illness and its ramifications. It seemed that if an underlying relationship problem existed previously, it was magnified by this stressful situation. In some cases, it triggered the disintegration of the family: “*I got sick [in Sweden] … and my ex’s reaction was so upsetting, that like, I got this kind of confirmation that we couldn’t be married anymore.*” (Mother 1). Of the 12 parents interviewed, four separated following their arrival back home.

As parents transitioned between health care systems, they often felt their needs were overlooked by health professionals both in Iceland and Sweden. The needs parents were overlooked varied, but mothers more than fathers talked about their psychological issues during and following their child’s hospitalization. Parents described how they felt alone in dealing with their health problems and their need for support: “*There is nobody thinking about the parents… I realized that there was no mental support provided abroad. And not here at home either.*” (Mother 8).

Parents’ vulnerabilities were expressed in their memories of distress. All mothers were recovering from childbirth when they traveled the first time with their infant to Sweden. Nevertheless, mother’s postpartum problems such as “*lactation mastitis*” (Mother 1), “*lactation suppression*” (Father 6), “*post-partum depression*” (Mother 3), and “*postpartum pain*” (Mother 12) were always secondary. Mothers described how they experienced physical pain and became sick following childbirth. Memories of this physical suffering were frequently associated with the traveling: “*I remember this extreme pain; I was walking down some long [airport] terminals*.” (Mother 1). This left first-time mothers feeling particularly vulnerable. Overall, parents were sleep deprived and tired during the child’s hospitalization period. However, the fatigue and emotional aftershock usually appeared after they arrived back home, following which, some mothers described they developed psychological problems: “*When she was eight months old, then I began experiencing traumatic stress and anxiety… It had been escalating since she came home.*” (Mother 3). For some, these health problems were only short-term, whereas in others they became long-term health issues.

#### 3.3.2. Feeling Overlooked: Culture and System

On their arrival in Sweden, parents had to adjust to a foreign environment, language, costumes, and regulations. In this situation, parents inevitably experienced cultural barriers. However, the parents’ ability to speak or understand English or Swedish was not assessed before they traveled to Sweden. These abilities were simply assumed.

Language barriers were the most common issues raised by parents. While most parents were able to communicate in English or Swedish, some were not. Those that were unable to speak English did not seek help from a translator. Indeed, the mere idea of a translator seemed awkward and even shameful; rather, parents described how they did their best or relied on other family members to help them: “*I’m very poor in English… but my mom and her boyfriend… helped me understand.*” (Mother 12).

Most parents felt welcomed during their stay in Sweden, although some mentioned cultural insensitivities from health professionals. In a few cases, parents noted that health professionals’ proficiency in English was lacking. This was surprising to parents and in retrospect they regretted not asking for better service.

Parents talked about how they felt that unrealistic expectations and responsibilities were being placed on them. Sometimes parents felt as if they were being taken for granted, even treated as employees: “*We were just in a part-time job, actually we were providing intensive care by watching over her the whole night.*” (Mother 3). Parents described how health professionals expected them to cope, under what they felt were unrealistic and extreme circumstances for them, that left them traumatized. These traumatic events were mostly related to their child’s suffering during medical procedures: “*My only memory is when a line was being removed, this was a long line, all the way into the heart. I just remember how much he screamed.*” (Mother 11).

When in Iceland, parents did not receive any follow-up support and there was no interdisciplinary team assigned to help the families. Parents were mostly left to navigate the systems and bureaucracy on their own to find help and support. They expressed surprise at the scarcity of resources available to them and described a gap in the system: “*Perhaps the biggest problem with the process is what happened afterwards. Because he died… We just came home and there was nobody that contacted us… We had to seek everything ourselves like psychological help and all that.*” (Father 6). Some parents were so concerned with these systemic flaws that they sought ways to help repair the system. These parents advocated for a multidisciplinary health care team to assist the whole family.

### 3.4. Finding Closure

The time span of parent’s situational transitions varied, but “*[getting] off the rollercoaster*” (Father 5) and finding closure was desired by all parents. Parents did not always describe a specific endpoint, but when they did it seemed fluid and sometimes attached to ‘ritual(s)’. The process of finding closure was expressed in surviving and compassion, with personal growth indicating a healthy transition.

#### 3.4.1. Surviving

All parents interviewed were survivors, they had been through tremendous challenges, stress, and even trauma, and were able to share this. The meaning parents placed on survival varied, however. For some, the stressful life event itself was their focus: “*It is a huge undertaking to go abroad. When I think back to this time, I just get shivers down my spine and if I had to do this again, I would have a nervous breakdown.*” (Mother 9). This was also exemplified in one father’s recollection of this time: “*we as parents, so often think back to this time with a sense of horror, it was a really dreadful time.*” (Father 4). In other cases, parents described this stressful experience as something they needed to endure in order to survive: “*I just wanted this to be over. Just to get through this and have it over with.*” (Mother 7).

Parents also described survival as a process of moving on and finding a new normal. This was illustrated by for example in growing their families or ending a spousal relationship. However, this process was perhaps most clearly illustrated by one father: “*Then you go off the rollercoaster and you just know: Wow! … but then you just keep going, just forward, and finally you start picking up the threads of a healthy life.*” (Father 5). In addition, recovering was also found in parents interviews as an indicator of closure: “*This is such a huge shock… recovering could’ve been much harder for us… but I think we’re just lucky.*” (Mother 7).

Parents who struggled the most were grieving some type of loss. To be able to move on, they had to reevaluate their experience and even reshape their memories. This was illustrated by one mother, whose daughter developed long term complications, in her statement that, “*my existence now is trying to make peace with my life… you know her difficult life. The life she will lead… trying to find meaning…*” (Mother 3).

#### 3.4.2. Showing Compassion 

Parents expressed their compassion for parents in the same situation as them, especially those who were more vulnerable. They questioned whether the current system was tailored for these specific groups and had concerns: “*[It] is absolutely necessary to ask people before they travel abroad [about English skills], because individuals with intellectual disabilities are having children for example, or parents who didn’t even finish elementary school and or aren’t good in languages. Then it is necessary… to have someone to translate for them.*” (Mother 9).

Following their experiences of traveling abroad, some parents joined peer-to-peer groups to support other parents and others even started lobbying within the Icelandic hospital system, for health care reforms: “*we have criticized the process, asked for changes…we are told a new protocol is in place…I just hope that if something like this happens again [child dies abroad], it goes better for those people.*” (Father 6).

Empathy-based survival guilt towards the suffering of other parents was also expressed by many parents. This empathy was directed towards other parents who either lost their child or had a much sicker child: “*After the funeral …the guilt I felt towards my sister- and brother-in-law. We’d just got through this and it went so well and then… their son just dies.*” (Mother 9).

## 4. Discussion

This study elicited parents’ experiences of their child’s CHD diagnosis, heart surgery abroad, and journey beyond. The overarching theme, living with the memories, highlighted the superimposition of this stressful live event on the expected transition to parenthood, where parents’ vivid memories still impacted their lives up to five years later. The most concerning finding was parents’ negative memories of the transition back home and their descriptions of roadblocks to recovery that followed. 

According to Meleis’s transitions theory [19], the constructs that define transitions are its nature (e.g., critical event), conditions (i.e., facilitators and inhibitors), and patterns of response (e.g., indicators of a healthy transition). Furthermore, all transitions are believed to build on universal commonalities, where disconnectedness is seen as the most pervasive one [19]. 

A parental transition triggered by child’s CCHD and need for heart surgery abroad has not been previously documented. However, other types of parental transitions focusing on child medical tourism [16,17,18] and hospitalizations of critically ill children [8,9,10] have been studied. Concurrently, our findings support the diverse and universal nature of transition experiences [19], although all parents experienced situational transitions, its nature—complexity, suddenness, degree of stress, and social support required —was particularly different. Thus, the uniqueness of our findings relates to the transitional conditions experienced by our parents.

Our findings showed both facilitators and inhibitors in parents’ environmental conditions. Connectivity represented itself both as a facilitator (e.g., in networks of health professionals and peer-groups) and an inhibitor (e.g., separation from child’s siblings). Separation or feeling separated is associated with personal, familial, socio-cultural, and geographical factors. However, separation from family and home life is hard on individuals in hospital settings and can lead to vulnerability, loneliness, and dependency [23]. 

The distance between home and hospital created a feeling of disconnection in parents in our study. Concurrently, others have shown that separation caused by geographical distance can negatively impact family relationships during child’s hospitalization. Specifically, parents’ likelihood of relationship difficulties with other family members tend to grow as the distance between hospital and home increases [24]. In our study, several parents described relationship tensions during their stay in Sweden, with some attributing this tension to the distance from home. Further, the separation from the child’s siblings at home was also hard on parents, and aligns with the findings of others [16]. Parents used modern technology (e.g., Facebook, Skype, Facetime) to connect with the left-behind siblings. This seemed however disjointed to their feeling of disconnectedness due to the physical distance. Another contributing factor may be siblings age, as all were relatively young, which might have made the utilization of technology perplexing.

In hospital settings, separation from home triggers a need for comfort and support that can be found through cultural connection. This includes seeking familiarity in terms of language and food, in addition to developing a sense of belonging in terms of social support networks [23]. Consistent with a meta-analysis and systematic reviews of parental coping with their child’s CHD [3], our participants used various coping strategies from surgery to childhood. However, their means of becoming culturally connected while abroad—particularly in the security, safety, and trust they placed in countrymen—is unique to our findings. Here, child’s grandparents and the health professionals working at the hospital abroad were of significance in creating this cultural connection.

Similar to that in other studies [3,25], grandparents were a significant source of support to parents in our study. Grandparents were a cornerstone in their habitat building, either caring for the child’s siblings at home or traveling with them abroad, thus creating familiarity through food and language. In hospital settings, such comfort can counterbalance negative emotions associated with being away from home and home life [23]. Importantly, the findings showed that the support provided by countrymen working as health professionals at the hospital abroad, created unique networks and relationships essential to the parents. These professionals provided parents with services ranging from direct medical care to assistance with practical matters, all seemingly done voluntarily. The shared culture and language appear to have created a feeling of safety, understanding, and familiarity, and a sense of belonging. Cultural competence in health care involves streamlining care and facilitating communication [26]. This could be done by matching patients and providers based on their cultural similarities [27,28]. Our results suggest that cultural competence in health care can be achieved by providing families traveling abroad with access to the medical care of health professionals who are fellow countrymen.

A key element facilitating healthy parental transitions from the PICU to a clinical ward is effective communication and information flow [8]. Furthermore, clarity in patient-provider communication is needed for facilitating and managing transitions, also imperative to securing quality care [29]. Our findings show that parents’ experienced communication challenges with health professionals while abroad, which amplified the imbalance of social capitals inherent in the client-providers encounters. The clearest communicational challenges reported related to language and other cultural barriers. Similar findings are reported in child medical tourism [16,17]. Although no participant asked for medical translator, they sought assistance from their fellow countrymen (i.e., family and health professionals) when needed. 

Language discrepancies between clients and health clinicians contribute to communication errors, especially if both speak a second language [30,31]. Furthermore, within these language-discrepant medical communication settings, individuals’ distress and vulnerabilities are likely to increase [30,32]. This is problematic and implies that assessing and anticipating families’ need for support prior to traveling (e.g., a medical translator) is necessary, as has been suggested by others [14]. It is therefore surprising that our participants and apparent health professionals did not anticipate this, even though both parties had to communicate in a second language (i.e., English). 

The transitions experienced by our parents following their child’s CHD diagnosis and cardiac surgery were extremely stressful. In a brief period, parents transitioned from expecting to go home with a healthy baby to having a critically ill newborn needing cardiac surgery abroad. Some even had to make this journey abroad three times. Parents’ descriptions of their aftershock, unmet needs, and short- and long-term health problems following their arrival back home, concur with the pediatric cardiac literature [6]. Similar findings are however not reported in the child medical tourism literature [16,17,18]. 

Personal growth and empowerment are a positive outcome of transitions [19], described by few parents in our study. This growth was best exemplified in the compassion and empathy parents showed toward their peers in similar situations, and in parents’ efforts to reform the current health care system. Concurrently, in a qualitative study, looking at parents mostly from low-income countries, majority of parents experienced personal growth (e.g., greater compassion for others) as a result of coming to the United States to seek medical treatment for their child [16]. In addition, a quantitative study [33] reported nearly 40% of parents experienced posttraumatic growth after their child’s admission to PICU. The different contexts and samples used in these studies compared to ours, may explain the inconsistency of experienced personal growth by our parents. Even so, all parents in our study described a path to recovery as they tried to fit the transition into their life story in a meaningful way. 

Our findings suggest an urgent need for follow-up care and support for this population, but currently, no such support is provided by health professionals in either Iceland or Sweden. A significant feature of transitional care is comprehensive and continued follow-up care for patients and families [19]. This is in line with pediatric cardiology guidelines [34] which recommend that a multidisciplinary health team supports children with CHD and their parents throughout their whole journey, including with follow-up care. Systematic reviews examining parents after their child’s PICU admission [35] and meta-analyses examining adult ICU survivors and their relatives [36] have indicated that follow-up appointments can improve mental health for these groups. These findings stress the urgency of offering the same follow-up service for families of children with CHD, particularly for at-risk groups such as those traveling abroad.

eHealth is considered a promising aid to lower psychiatric morbidity in families of children with CHD [34,37] and is particularly relevant for the population of the current study. This mode of health care creates opportunities for interactive communication between all stakeholders, allowing health care teams at home and abroad to collaborate more easily with parents as they transition between systems. Furthermore, via website apps, psychological follow-up care could, for example, permit parents to make virtual revisits to the hospital abroad.

This study has both limitations and strengths for consideration. Of the seven mothers and five fathers who participated, only eight children with CHD were represented. The range of possible experiences might thus have been restricted because both parents could participate. However, parent couples’ experience of a stressful event often varies greatly [38]. A written request was sent to 25 parents via e-mail. Fifteen parents replied to the e-mail, but only 12 parents participated in the study. It is unclear whether the 13 parents who did not participate are in some way demographically different from those who did. The nearly equal number of mothers and fathers, and the wide range of sociodemographic characteristics in the participant sample, however, strengthened the study findings. Additionally, the primary categories reappeared in all of the interviews, thus creating a saturation that strengthened the results. The study findings may not be generalized to other populations, but may be transferable to other parents of children with CHD traveling abroad for cardiac surgery. The sample size was relatively small. However, the fact that similar sample sizes have been used in other studies [39,40] and the emphasis on richness rather than sample size in content analysis [41] provides strength to our findings. The trustworthiness of the study findings lies in the methods used and the experience of the three authors. All authors are registered nurses with pediatric clinical experience. The second author (A.S.S.) has extensive experience with pediatric acritical care cardiology and working with parents of children with CCHD. Although all authors are experienced researchers, the second author also has extensive experience in qualitative content analysis methods.

## 5. Conclusions

The lived experiences of parents when their child underwent complex heart surgery abroad are filled with difficult memories and emotions. Parents’ readiness and need for support was heightened after arriving home from abroad. However, these needs were not met by either of the health care systems involved. Culturally competent care for the families must be designed and implemented, starting with an assessment of language skills prior to the travel. Assigning families to multidisciplinary health care teams and providing them with follow-up support are necessary. Here, the use of eHealth interventions to improve interactive communication between all stakeholders is promising.

## Figures and Tables

**Table 1:** Family demographics and background (*n* = 12).

Parent Information	*n* (%)
Role	
Mother	7 (58)
Father	5 (42)
Age ^a^	
20–29 years	5 (45)
30–39 years	5 (45)
40–49 years	1 (10)
Marital status ^a^	
Married/common law	7 (63)
Living separately	3 (27)
Divorced	1 (10)
Education	
Highschool diploma or vocational education	5 (42)
Tertiary (collage/university)	5 (42)
Advanced college/university	2 (16)
Total monthly family income ^a^	
≤789,000 ISK	4 (37)
790,000–1.29 million ISK	5 (45)
≥1.3 million ISK	2 (18)
Other children at the time (no)	8 (67)
Both parents traveled abroad (yes)	12 (100)
Relatives traveled abroad with parents (yes)	8 (67)
Length of stay abroad ^a^	
≤14 days	4 (36)
15–21 days	5 (46)
>22 days	2 (18)
Multiple travels abroad (no)	9 (75)
Time from travel abroad to study ^a^	
1–2 years	5 (45)
3–5 years	6 (55)
**Child Information**	***n* (%)**
Gender (boy)	10 (83)
Age of child at first travel abroad	
1–10 days old	7 (58)
11–30 days old	3 (25)
>31 days old	2 (17)
Delivery type (vaginal)	7 (58)
Time of diagnosis (postnatal)	9 (75)
Diagnosed heart defect (ICD-10) ^b^	
Congenital malformations of great arteries (Q25)	6 (50)
Congenital malformations of pulmonary and tricuspid valves (Q22)	2 (17)
Congenital malformations of cardiac septa (Q21)	3 (25)
Discordant ventriculoarterial connection (Q20.3)	1 (8)

^a^ Demographic data missing for one participant. ^b^ Category based on the International Classification of Diseases, Tenth Revision.

**Table 2:** Selected interview questions.

Could you tell me what it was like learning that your child needed heart surgery abroad? Could you please tell me what it was like while you were waiting to travel abroad for your child in surgery? Could you please tell me what it was like traveling abroad for your child surgery abroad? Could you tell me what it was like while you were waiting for your child in surgery? Could you tell me how you experienced the hospital stay abroad? Could you tell me how you experienced the support provided by the health care staff? Could you tell me what it was like being away from your family (e.g., other children)? Could you tell me about the things that made this experience stressful for you? Could you tell me about what helped you during stressful time periods? Could you tell me your experiences with the discharge process? Could you tell me how this experience has affected your health? Relationships? Could you tell me your experience of flying with your child back home? Could you tell me how it was like coming back home after the surgery? Could you please describe your life after you were discharged from the hospital abroad and back home?

**Table 3:** Overarching theme, main categories, and subcategories.

Overarching Theme: Living with the Memories	
Categories	Subcategories
1. Being in an unknown situation	a. Disrupted life
	b. Life on hold
2. Feeling connected	a. Supportive environment
	b. Handling the experience
3. Wishing being accepted	a. Parenting roles and needs
	b. Feeling overlooked: Culture and system
4. Finding closure	a. Surviving
	b. Showing compassion

## Supplementary Materials

The following are available online at https://www.mdpi.com/1660-4601/17/23/8840/s1, Table S1: COREQ checklist, Table S2: Coding tree.
