# Supplementary material for: Living with the Memories—Parents’ Experiences of Their Newborn Child Undergoing Heart Surgery Abroad: A Qualitative Study
Source: Int J Environ Res Public Health. 2020 Nov 28;17(23):8840. doi: 10.3390/ijerph17238840 (PMC7730968; doi:10.3390/ijerph17238840)
Supplement: Supplementary file 1 [file ijerph-17-08840-s001.zip › Supporting File 2- table S2.docx]

**Table S2:** Coding Tree: Codes for categories and subcategories

| **BEING IN AN UNKNOWN SITUATION** | **FEELING CONNECTED** | **WHISHING BEING ACCEPTED** | **FINDING CLOSURE** |
| --- | --- | --- | --- |
| *Disrupted life &Life on hold* | *Supportive environment & Handling the experience* | *Parenting roles and needs & Feeling overlooked* | *Surviving & Showing compassion* |
| Shock | Not knowing | Being included | Cardiologist |
| Travel | Mistrust | Being informed | Icelanders |
| Change (roller-coaster) | Insecure | Being trusted | Countrymen |
| Unexpected | Lost | Prepared | Grandparents/family |
| Stressors (critical incident) | Estranged | Breastfeeding | Other parents |
| Aftershock | Frustrated | Burden of caring | Neistinn/Spark |
| Home away from home | Afraid | Understood | Ronald McDonald house |
| Home | Uncertainty | Maternal | Empower/help other parents |
| Visiting | Separation | Needs (physical, mental, social) | Guilt |
| Timing (good, bad, future, unknown) | Isolated | Acceptance as foreigner | Thankful |
| Pace (fast, slow) | Stressed | Welcomed | Glad |
| Staying | Worry | Language | Embracing memories |
| Relationships | Regret | Barriers | Recovering |
| Parenthood (shared) | Not remembering | Unrealistic responsibilities | Moving on |
| Connecting | Strained | Breaking the rules |  |
| Communicating | Suffering | Professional service |  |
| The news | Symptoms (physical, mental, social) | Not belonging |  |
| Going abroad | Aftershock | Aftercare |  |
| Surgery | Supported | Limitations |  |
| The phone-call | Trusting | System |  |
| Seeing child after surgery | Caring | Culture |  |
| Discharge (PICU, Cardia Ward, home) | Security | Role |  |
| The/a visit | Closeness | Need |  |
| Spouse | Attached | Parenting |  |
| Going home | Being able (own strength) | Overlooked |  |
| Reuniting | Certainty |  |  |
| For recovery | Recovering |  |  |
| A Better life (hoping) | Resilience |  |  |
| Envisioning (pictures, visualize) | Blocking memories |  |  |
| Retrieval | Catastrophize |  |  |
| Stories | Offloading |  |  |
| Conversations | Listening |  |  |
| Interactions with others (PICU, airplane) | Adjusting/Adapting |  |  |
| Distressful memories | Accepting help |  |  |
|  | Seeing own uniqueness |  |  |
|  | Fleeing |  |  |
|  | Thanking |  |  |
|  | Information |  |  |
|  | Knowledge |  |  |
|  | Understanding |  |  |
|  | Believing |  |  |
|  | Learning |  |  |
|  | Preparation |  |  |
|  | Neistinn/Spark (webpage, facebook) |  |  |
|  | Other parents |  |  |
